# Comparative Study of Drought Stress Effects on Traditional and Modern Apple Cultivars

**DOI:** 10.3390/plants10030561

**Published:** 2021-03-16

**Authors:** Ines Mihaljević, Marija Viljevac Vuletić, Domagoj Šimić, Vesna Tomaš, Daniela Horvat, Marko Josipović, Zvonimir Zdunić, Krunoslav Dugalić, Dominik Vuković

**Affiliations:** Agricultural Institute Osijek, Južno predgrađe 17, HR-31000 Osijek, Croatia; marija.viljevac@poljinos.hr (M.V.V.); domagoj.simic@poljinos.hr (D.Š.); vesna.tomas@poljinos.hr (V.T.); daniela.horvat@poljinos.hr (D.H.); marko.josipovic@poljinos.hr (M.J.); zvonimir.zdunic@poljinos.hr (Z.Z.); krunoslav.dugalic@poljinos.hr (K.D.); dominik.vukovic@poljinos.hr (D.V.)

**Keywords:** chlorophyll, lipid peroxidation, OJIP test, photosynthesis, proline, water content

## Abstract

Genotype-dependent responses of apples to drought stress were evaluated between commercial and traditional apple cultivars. The results indicate different mechanisms of tolerance to investigated drought stress conditions. Chlorophyll fluorescence induction (OJIP) parameters, chlorophyll and carotenoid content, malondialdehyde (MDA), hydrogen peroxide (H_2_O_2_), proline, phenols and leaf water content (WC) were measured. The traditional cultivar “Crvenka” confirmed the best tolerance to a drought stress condition, presenting higher photosynthetic efficiency, higher leaf water content, higher levels of chlorophyll content and lower lipid peroxidation with greater membrane stability. The commercial cultivar “Golden Delicious Reinders” showed decreased water content in leaves, increased lipid peroxidation levels and photoinhibition. Considering all results, the commercial cultivar “Golden Delicious Reinders” was adversely affected by drought, while traditional cultivars exhibited better tolerance to drought stress.

## 1. Introduction

In recent years, numerous studies have been published about the effects of global climate change on ecosystems, and it is predicted that climate change will cause extreme temperatures and droughts. Drought has become a major abiotic stress factor that adversely effects plant growth, survival and limits crop productivity [1], causing a reduction in fruit yield and fruit quality [2]. It has been shown that water deficit influences various physiological, biochemical, metabolic and molecular processes in various plants, including apple trees [3,4]. Lack of water in plants induces oxidative stress [5,6], overproduction of reactive oxygen species (ROS), including the superoxide radical (O_2_^−^) and hydrogen peroxide (H_2_O_2_), which cause lipid peroxidation and damages the membrane, proteins, chlorophyll, nucleic acids and cell death [7,8]. Drought can cause a significant reduction and damage in photosynthesis and chlorophyll degradation [9,10]. According to Chaves et al. [11], the negative effects of drought on plant physiology are dependent on the intensity and duration of the drought stress and the genetic capacity of the plant to cope and survive in this stress conditions. To cope with drought stress and protect themselves from oxidative stress, plants have evolved antioxidant defense mechanisms including antioxidant enzymes (e.g., peroxidase (POD), superoxide dismutase (SOD), catalase (CAT)) and non-enzymatic antioxidants (e.g., phenolic, ascorbic acid, glutathione, carotenoids) [12,13,14]. Some plants accumulate osmolytes such as proline, glycine betaine and soluble sugars to protect themselves and to alleviate the drought stress condition [15,16,17]. Recent studies reported that some secondary metabolites synthesized in plant organs such as volatile compounds terpens [18] and some phytohormones such as brassinolide [19] alleviated the effect of drought stress on plants by improving the plant’s defense system. The apple (*Malus domestica* Borkh.), is one of the most economically important continental fruits worldwide and one of the most consumed fruits in the world [20]. It is predicted that in the future, different regions in Europe will be affected by drought [21]; therefore, it is necessary to prepare apple production for stronger droughts. The ability of apple trees to cope with drought stress and to achieve a high yield in these conditions will be of great economic importance. Proper selection of drought resistant cultivars and rootstock is one of the strategies to reduce the impact of this stress and contribute to a more stable apple production. The apple is the fruit that is most commonly cultivated in Croatia, but in intensive apple production they are mostly represented by commercial, modern cultivars with high productivity. Modern cultivars are foreign, high yielding cultivars, which do not represent the diversity of local conditions. They were derived from plant breeding. Traditional cultivars are those cultivars that have been related to specific region, mostly grown in backyards and small orchards whose origin is mostly not known. They are locally adapted to its natural environment. Due to global climate change, the most important characteristics of plants is their tolerance to different abiotic and biotic stresses such as drought, extreme temperature, disease and pest resistance. These characteristics are usually present in traditional cultivars, but because of the great representation of high yielding commercial cultivars, interest in the production of traditional cultivars has decreased. In recent years, there has been a great interest in growing traditional cultivars that are suitable for production in this area [22]. In previous research, traditional cultivars have shown that they have very valuable fruits, contain more polyphenols [23], more fibers, proteins, sugars, β-carotene and vitamin E [24], and they have a better capacity to tolerate biotic and abiotic stress [25] compared to commercial cultivars. The aims of this study were (1) to elucidate the adaptation mechanisms of two traditional apple cultivars to drought-stressed conditions in comparison with commercial cultivar; (2) to obtain information about the genetic potential of drought stress tolerance of these traditional cultivars for the future production and breeding of new apple cultivars. Measuring chlorophyll *a* fluorescence has been widely used as a non-destructive method to analyze the structure and function of the photosynthetic apparatus, particularly PS II, under different abiotic stresses, including drought stress [26,27,28]. In this study we investigated the drought tolerance of selected apple cultivars by OJIP (chlorophyll *a* fluorescence analysis). For a better understanding of the defense mechanisms of investigated cultivars, beside photosynthetic efficiency, we studied lipid peroxidation, proline, phenol, chlorophyll and water content in the leaves. To the best of our knowledge, the drought stress tolerance of these traditional apples has not been previously studied.

## 2. Results

### 2.1. Effect of Drought Stress on Chlorophyll Fluorescence Parameters

**Figure 1:**
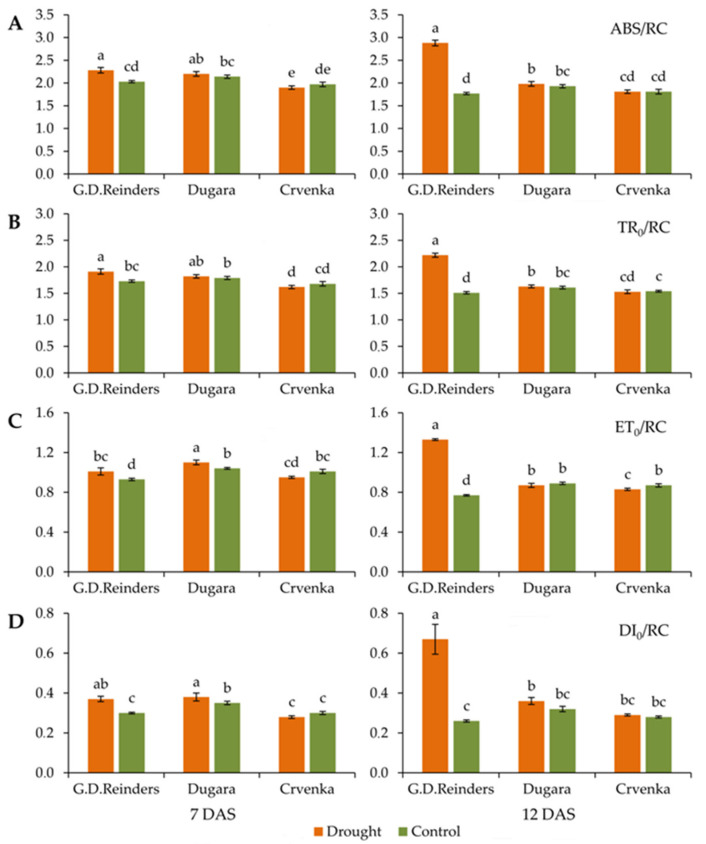
Absorption per active reaction center (ABS/RC) (**A**), trapped energy flux per active reaction center (TR_0_/RC) (**B**), electron transport flux per active reaction center (ET_0_/RC) (**C**), dissipation energy per active reaction center (DI_0_/RC) (**D**), measured in control and drought-stressed apple cultivars (“Golden Delicious Reinders”, “Crvenka” and “Dugara”) at 7 and 12 DAS (day after stress). Values are presented as relative units. Values are mean ± SE (*n* = 15). Different letters indicate significant difference among treatments and cultivars at *p* < 0.05 according to the LSD test.

Drought tolerance of three apple cultivars (two traditional and one commercial) was evaluated through analyzing several fluorescence parameters. Significant increase in absorption per active reaction center (ABS/RC), trapped energy flux per active reaction center (TR_0_/RC), electron transport flux per active reaction center (ET_0_/RC) and dissipation energy per active reaction center (DI_0_/RC) at 7 and 12 DAS (day after stress) between control and drought-stressed plants were observed in cultivar “Golden Delicious Reinders” (Figure 1A–D). The increase in the following parameters were more pronounced at 12 DAS: absorption per active reaction center (ABS/RC) (value was higher by 63% compared to control plants) (Figure 1A), trapped energy flux per active reaction center (TR_0_/RC) (value was higher by 47% compared to control plants) (Figure 1B), electron transport flux per active reaction center (ET_0_/RC) (value was higher by 72% compared to control plants) (Figure 1C), dissipation energy per active reaction center (DI_0_/RC) (value was higher by 157% compared to control plants) (Figure 1D). Traditional cultivar “Crvenka” showed no significant differences between control and drought treatment plants in the previously mentioned parameters, while “Dugara” showed significant differences for ET_0_/RC and DI_0_/RC at 7 DAS (Figure 1A–D).

**Figure 2:**
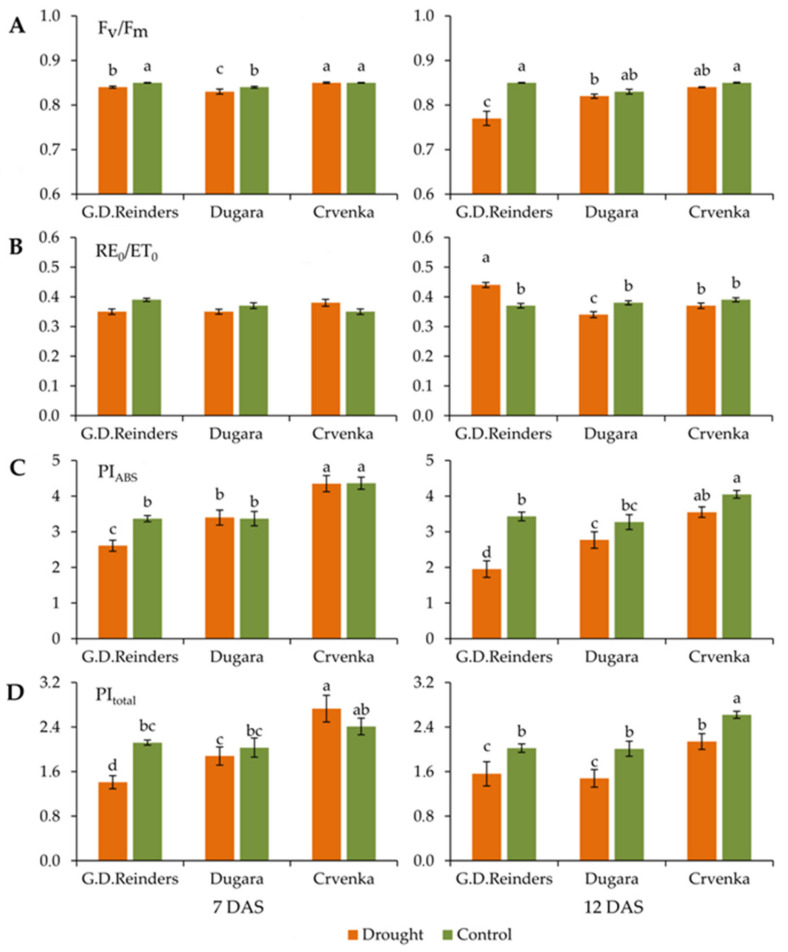
Maximum quantum yield of photosystem II (F_v_/F_m_) (**A**), efficiency/probability with which an electron from the intersystem electron carriers transferred to reduce end acceptors at the PSI acceptor side (RE_0_/ET_0_) (**B**), performance index on absorption basis (PI_ABS_) (**C**), performance index for energy conservation from exciton to the reduction of PSI end acceptors (PI_total_) (**D**), measured in control and drought-stressed apple cultivars (“Golden Delicious Reinders”, “Crvenka” and “Dugara”) at 7 and 12 DAS (day after stress). Values are presented as relative units. Values are mean ± SE (*n* = 15). Different letters indicate significant differences among treatments and cultivars at *p* < 0.05 according to the LSD test.

Significantly lower values in drought-stressed plants were observed in “Golden Delicious Reinders” for a maximum quantum yield of photosystem II (F_v_/F_m_) and performance index on absorption basis (PI_ABS_) (Figure 2A,C). Decreases in these parameters were also more pronounced at 12 DAS: maximum quantum yield of photosystem II (F_v_/F_m_) (value was lower by 10% compared to control plants) (Figure 2A), performance index on absorption basis (PI_ABS_) (value was lower by 75% compared to control plants) (Figure 2C). There were no statistical differences in these parameters, between control and drought-stressed plants in the leaves of traditional cultivars “Crvenka” and “Dugara” (Figure 2A,C). Values of efficiency/probability with which an electron from the intersystem electron carriers is transferred to reduce end electron acceptors at the PSI acceptor side (RE_0_/ET_0_) did not change significantly after 7 days of drought in all cultivars. After 12 days of drought treatment, the value of (RE_0_/ET_0_) in “Golden Delicious Reinders” was increased, while in cultivar “Dugara” it was decreased. The values of this parameter did not change in cultivar “Crvenka” even after 12 DAS (2B). However, the results showed that the highest values of (PI_ABS_) were recorded in cultivar “Crvenka” after 7 days of drought (4.35) and 12 days of drought (4.05) (Figure 2C). Similarly, under the drought stress, PI_total_ values showed the highest drop in the leaves of “Golden Delicious Reinders”; values were significantly lower than those in non-stressed plants. The smallest decrease in this parameter was recorded for cultivar “Crvenka” (Figure 2D).

### 2.2. OJIP Curve

**Figure 3:**
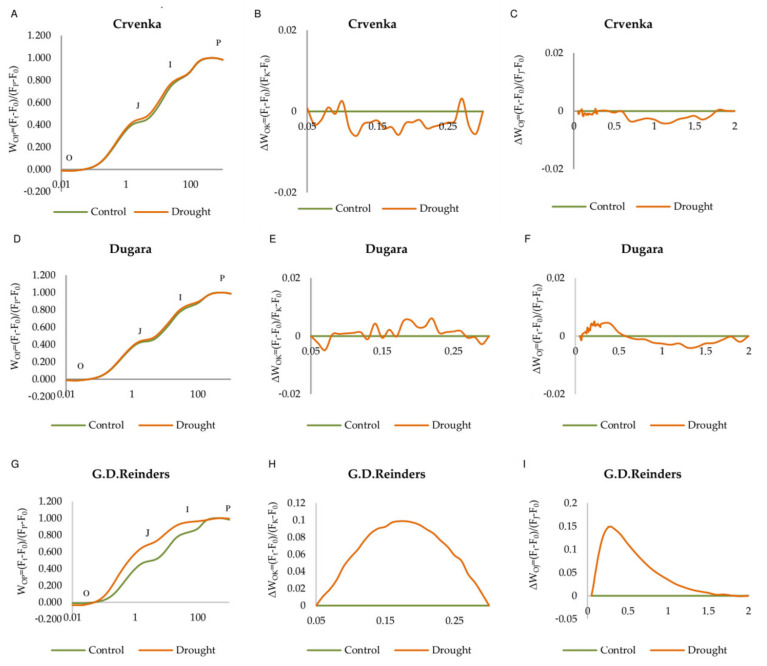
Chlorophyll a fluorescence OJIP transient curves of leaves of three apple cultivars submitted for 12 days to drought stress. Transients curves were normalized between O and P steps: W_OP_ = (F_t_ − F_0_)/(F_P_ − F_0_) (**A**,**D**,**G**). The difference kinetics ΔW_OJ_ (**C**,**F**,**I**) reveal the K-band; ΔW_OK_ (**B**,**E**,**H**) reveals the L-band. ΔW_OJ_ [ΔW_OJ_ = V_OJ(treatment)_ − V_OJ(control)_] and ΔW_OK_ [ΔW_OK_ = V_OK(treatment)_ − V_OK(control)_] were calculated from the comparisons of the stressed and control plants. Mean values (*n* = 15).

The fluorescent transient OJIP curves represent a plot of fluorescence data for each cultivar plotted on logarithmic time scale at 12 days of drought treatment. They revealed distinct differences between cultivars and treatments. In drought treatments, the change of the OJIP curve of traditional apple cultivars “Crvenka” and “Dugara” was less visible than that commercial cultivar “Golden Delicious Reinders”. Our results showed that drought treated plants of the “Golden Delicious Reinders” cultivar had higher fluorescence intensity at the J step (2 ms) and I step, compared to traditional cultivars (Figure 3A,D,G), resulting in a dramatically changed OJIP curve shape. Drought stressed plants of “Dugara” cultivar exhibited slightly positive L band, with small amplitude (Figure 3E). We also recorded positive L and K bands in “Golden Delicious Reinders”, with much more pronounced L and K band amplitude (Figure 3H,I). The traditional cultivar “Crvenka” was observed with a negative L and K bands (Figure 3B,C).

### 2.3. Chlorophyll and Carotenoid Content

**Figure 4:**
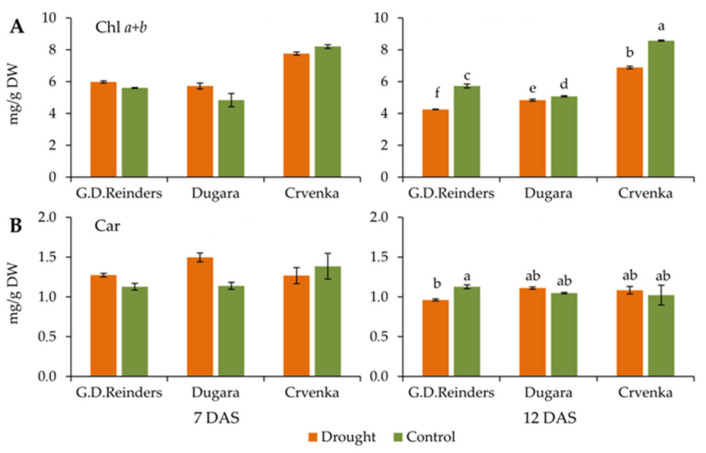
Total chlorophyll (Chl *a + b*) (**A**) and carotenoid (Car) (**B**) contents measured in control and drought-stressed apple cultivars (“Golden Delicious Reinders”, “Crvenka” and “Dugara”) at 7 and 12 DAS (day after stress). Values are mean ± SE (*n* = 5). Different letters indicate significant difference among treatments and cultivars at *p* < 0.05 according to LSD test.

The concentration of total chlorophyll content remained unchanged in drought-stressed leaves of all cultivars at 7 DAS compared to control (Figure 4A). All apple cultivars after 12 day of drought showed a significant decrease in total chlorophyll content, compared to control. Among the plants subjected to drought stress, cultivar “Crvenka” retained the highest chlorophyll content at 7 DAS (7.76 mg/g DW) and at 12 DAS (6.88 mg/g DW) (Figure 4A). Leaf carotenoids content of all apple cultivars under drought did not differ from control at 7 and 12 DAS except in cultivar “Golden Delicious Reinders” at 12 DAS (value was lower 18% compared to control plants) (Figure 4B)

### 2.4. MDA and H_2_O_2_ Content

**Figure 5:**
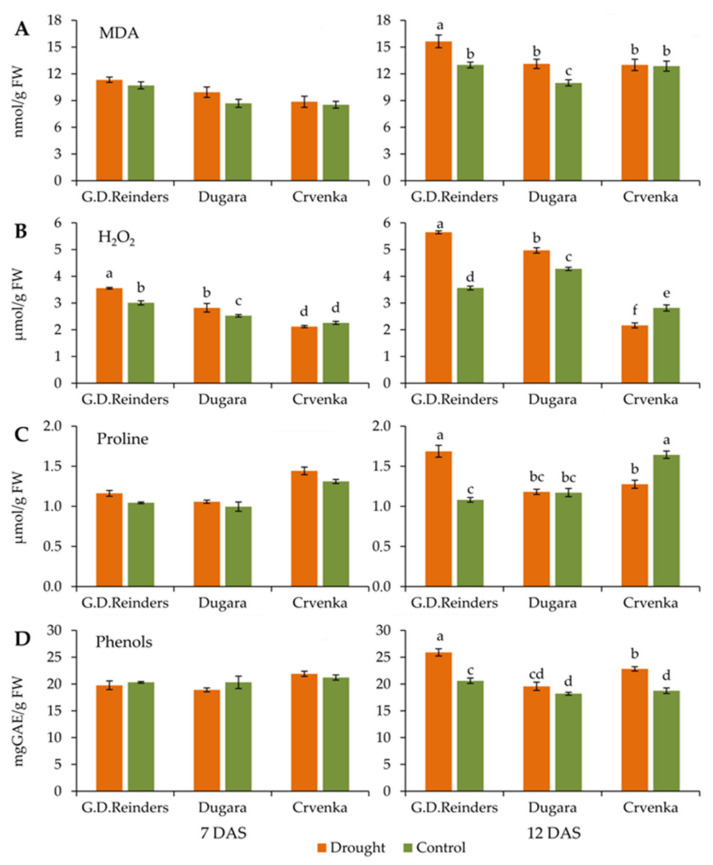
Malondialdehyde (MDA) (**A**), hydrogen peroxide (H_2_O_2_) (**B**), proline (**C**) and phenols (**D**) contents measured in control and drought-stressed apple cultivars (“Golden Delicious Reinders”, “Crvenka” and “Dugara”) at 7 and 12 DAS (day after stress). Values are mean ± SE (*n* = 5). Different letters indicate significant differences among treatments and cultivars at *p* < 0.05 according to the LSD test.

Lipid peroxidation was determined by evaluating the malondialdehyde (MDA) content in leaf tissues. In our study, the MDA significantly increased only at 12 DAS in cultivars “Golden Delicious Reinders” and “Dugara”, while the MDA content in the leaves of cultivar “Crvenka” remained unchanged (Figure 5A). The highest accumulation of MDA under drought treatment was observed in cultivar “Golden Delicious Reinders” at 12 DAS (15.19 nmol/g FW) (Figure 5A). A significant increase in H_2_O_2_ content after 7 and 12 DAS compared to control was detected in “Golden Delicious Reinders” and “Dugara” cultivars (Figure 5B). “Crvenka” showed significantly lower accumulation of H_2_O_2_ in drought-stressed plants compared to control for 30% only after 12 DAS (Figure 5B). After 7 and 12 DAS, cultivar “Golden Delicious Reinders” was observed to have the highest accumulation of H_2_O_2_ (3.55 µmol/g FW and 5.6 µmol/g FW).

### 2.5. Proline and Phenols Content

After 7 days of drought, the contents of proline remained unchanged in all cultivars, as compared with the corresponding controls (Figure 5C). The contents of proline at 12 DAS significantly increased under drought stress only in “Golden Delicious Reinders”. There were no statistical differences, between control and drought stress plants in cultivar “Dugara”, while in cultivar “Crvenka” a significant decrease in proline content was recorded (Figure 5C). Phenols content in the leaves of all apple cultivar did not change at 7 day, compared to control (Figure 5D). After 12 days of treatment drought stress induced an accumulation of phenols content in “Golden Delicious Reinders” and “Crvenka” cultivars. A significantly higher phenols content was detected in “Golden Delicious Reinders” (25.8 mg GAE/g FW) compared to “Crvenka” (22.82 mg GAE/g FW). There were no statistical differences between control and drought stress plants in cultivar “Dugara” (Figure 5D).

### 2.6. Water Content in the Leaves

**Figure 6:**
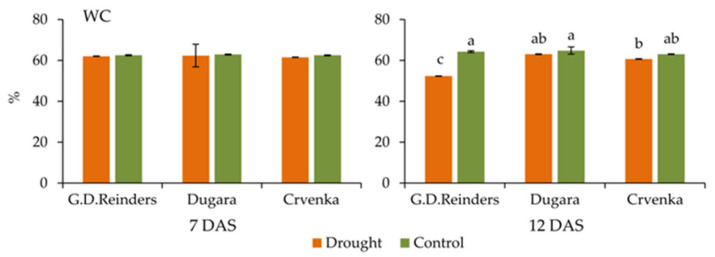
Water content in leaves measured in control and drought-stressed apple cultivars (“Golden Delicious Reinders”, “Crvenka” and “Dugara”) at 7 and 12 DAS (day after stress). Values are mean ± SE (*n* = 3). Different letters indicate significant differences among treatments and cultivars at *p* < 0.05 according to the LSD test.

There were no significant differences in leaf water content between cultivars under control and drought condition at 7 DAS. The water content (%) in the leaves of the studied apple cultivars decreased significantly only in cultivar “Golden Delicious Reinders” after 12 day of drought treatment (value was lower 23% compared to control plants) (Figure 6).

## 3. Discussion

Some research has been conducted on the fruit quality characterization of traditional cultivars in Croatia and Balkan Region [29,30]. The molecular analyses were also conducted on these cultivars and revealed a clear differentiation between traditional and commercial cultivars [31], but no information is available about the effects of abiotic stresses on them. In this study we evaluated and compared some physiological and biochemical traits, in two traditional cultivars and one commercial cultivar, under a short period of drought, to understand their tolerance to drought stress.

Based on the chlorophyll fluorescence parameters, we investigated and compared the changes in fluorescence characteristics of PS II of selected cultivars under drought stress. The present study showed significant differences in leaf photochemistry of investigated cultivars, by analysis of OJIP kinetics. Our results showed that drought treated plants of “Golden Delicious Reinders” have higher fluorescence intensity at the J phase and I phase compared to “Dugara” and “Crvenka”, indicating a stronger decline and inhibition in electron transport beyond the Q_A_ and beyond PQH_2_ [32,33]. Simultaneously, the appearance of the positive L peak (around 120–150 μs) and K peak (around 300 μs) was observed in the OJIP transient of that cultivar. According to Strasser and Stirbet [34], the L band is an indicator of energetic connectivity or grouping between PS II units and positive L band reflects lower connectivity between PSII units. On the other hand, negative L band points on higher connectivity, efficient use of the excitation energy and higher stability of the photosynthetic system [35]. Data on the L-band indicate that in “Golden Delicious Reinders” and “Dugara”, drought stress treatment resulted in a decrease in energetic connectivity, while the cultivar “Crvenka” showed an increase in energetic connectivity. Similarly, the negative L-band occurred in the leaves of transgenic rice (overexpressing the *OsNAC10*-improved drought-stress tolerance) compared to non-transgenic under drought condition [36]. When plants are exposed to stressful conditions, the K-band occurs within the 200–300 μs range of the ChlF induction curve which has been associated with an inhibition and inactivation of the oxygen-evolving complex (OEC) [37]. In this study, the appearance of K-band suggested that drought in the leaves of “Golden Delicious Reinders” caused an inactivation of the OEC, while cultivar “Crvenka” retained a stable OEC (negative K band). L and K-bands with positive amplitudes were previously recorded for passion fruit [38] and sunflower [39] when exposed to drought. Drought significantly affected all the photosynthetic parameters studied in this study. Higher average absorption (ABS/RC), trapping (TR_0_/RC), electron transport (ET_0_/RC) and dissipation (DI_0_/RC) in the drought-stressed plants of “Golden Delicious Reinders” indicate inactivation of a certain part of RCs, due to inactivation of OEC, as well as the transformation of active RCs to silent [40]. We supposed that the greater number of inactive reaction centers was the basic reason for the higher dissipation of absorbed light. The inactivation of RCs due to drought stresses was evidenced by the decline in F_v_/F_m_. Similar behaviour of these parameters under drought-stressed grape leaves has been reported by Wang et al. [41]. Boguszewska-Mańkowska et al. [42] noticed an increase in the parameters ABS/RC, DI_0_/RC and TR_0_/RC and a large decrease in PI_ABS_ in drought-sensitive potato cultivars subjected to drought. In traditional cultivar “Crvenka” the values of these parameters remained unchanged under the influence of drought which indicated that the photosynthetic apparatus of cultivar “Crvenka”, with the highest values of PI_ABS_, was the most tolerant to investigated drought condition. PI_total_ was used to measure the performance up to the reduction of PSI end electron acceptors and its values represent the energy flow efficiency of the photosynthetic transport chain beyond PS II [40]. This parameter is the product of the performance index on absorption basis PI_ABS_ and the probability that an electron can move from reduced intersystem electron acceptors to PSI end electron acceptors (RE_0_/ET_0_). There was no difference in RE_0_/ET_0_ between drought-stressed and control plants in tolerant cultivar “Crvenka”, indicating that the electron flow was proceeding normally from PQH_2_ to the PSI end electron acceptors. The highest values of parameter PI_total_ also confirmed the better performance of PSI acceptor side in traditional cultivar “Crvenka” as compared to “Dugara” and “Golden Delicious Reinders” under drought stress. Increase of RE_0_/ET_0_ in the drought treatment in “Golden Delicious Reinders” is probably due to decrease in redox balance of PSII electron acceptors because of lower PSII activity. The parameter RE_0_/ET_0_ decreased by drought only in traditional cultivar “Dugara”. According to Schansker et al. [43], reduced parameter RE_0_/ET_0_ indicated that drought had a negative effect on electron flow at the acceptor side of PSI caused by an inactivation of ferredoxin-NADP^+^-reductase. The lower value of these parameter in “Dugara” indicate unsuitable electron transfer between two photosystems and that the acceptor side of PS I might be inhibited. This was reflected in the lower level of the PI (_total_) in this cultivar. The effects of drought stress observed in parameters PI (_total_) and RE_0_/ET_0_ are in accordance with a previous report of Jia et al. [44] in drought-stressed maize plants. (F_v_/F_m_) known as maximum quantum yield of PS II represent the efficiency of PSII primary photochemistry. Decreased value of that parameter under 0.75 indicates that PS II has been damaged [45]. Decrease of this parameter was observed only in the leaves of commercial, drought sensitive cultivar, “Golden Delicious Reinders”. This finding is consistent with the previous report of Faraloni et al. [46] who showed that the the F_v_/F_m_ values decreased in the sensitive cultivars of olive, whereas the tolerant cultivar did not show any decrease in F_v_/F_m_. In this study, we found that F_v_/F_m_ recorded values of all three cultivar were in the range of 0.77–0.85. Despite the fact that the OJIP curves obviously showed damage to the OEC complex and lower energetic connectivity between PS II units of the “Golden Delicious Reinders”, the values of F_v_/F_m_, although they were significantly decreased, were not below 0.75. However, parameter F_v_/F_m_ is a well-known indicator of stress; this result indicates that drought stress has relatively little effect on the parameter F_v_/F_m_ in this study. Previous reports also suggest that drought has relatively slight effect on the parameter F_v_/F_m_; thus, it is not a sensitive parameter for analysing drought stress [41,47].

Based on the obtained results we supposed that after 12 days of drought stress, decreasing trends of PI_ABS_ and PI_total_ values of the commercial cultivar “Golden Delicious Reinders” were mainly due to an inactivation of PS II RCs, a higher increase in TR_0_/RC and thereby ABS/RC, ET_0_/RC and DI_0_/RC. While in cultivar “Dugara”, the most sensitive components of the photosynthetic electron transport chain seemed to be the probability that an electron from the intersystem electron carriers is transferred to reduce end electron acceptors at the PSI acceptor side RE_0_/ET_0_. The highest photosynthetic activity in “Crvenka” was due to the highest amount of active PSII reaction centers and stable absorption flux. Our results indicated that the photosynthetic apparatus of traditional cultivar “Crvenka” was more tolerant to drought stress than “Golden Delicious Reinders” and “Dugara”.

Chlorophylls as the main photosynthetic pigments in plant leaves reflect the photosynthetic capacity of leaves and overall plant vitality. The chlorophyll content of plant leaves is considered as a good stress indicator for evaluating tolerance levels of various crops to different abiotic stresses, including drought stress [48]. Previous research documented that drought stress reduced the accumulation of chlorophyll content in apple rootstock [49,50]. Our research findings show that in drought treated plants chlorophyll content was also decreased, but only after 12 DAS. The decreases were more pronounced in the commercial cultivar “Golden Delicious Reinders”, while in “Crvenka” the decreases were lower. These results are in agreement with research of Bhusal et al. [10] where degradations in total chlorophyll content were lower in the more tolerant “Fuji” apple than in “Hongro” apples. Carotenoids play essential roles in photosynthesis, since they protect photosystem II from photo-oxidative damage [51]. Khoyerdi et al. [52] reported that the pistachio cultivar with higher carotenoids content was more tolerant to drought stress. In the present study, in contrast to decreases in the total chlorophyll content, the carotenoid content of traditional cultivars “Dugara” and “Crvenka” remained stable. After 12 DAS, total carotenoids were reduced only in commercial cultivar “Golden Delicious Reinders”, probably because the xanthophyll cycle was not activated properly to protect PS II from photoinhibition [53].

Decreased PS II activity under drought is connected with oxidative stress and cell membrane injury caused by increased lipid peroxidation [54]. MDA is a product of lipid peroxidation, often used as a measure of oxidative stress during drought stress [55]. Different abiotic stresses including drought induce the production of different kinds of reactive oxygen species (ROS) such as H_2_O_2_, which damage the membrane lipids [56]. Our results showed that higher MDA concentration in drought-stressed plants was associated with higher H_2_O_2_ content. The significant increase in MDA and H_2_O_2_ contents with drought stress progressed in the two apple cultivars “Golden Delicious Reinders” and “Dugara” suggested that drought stress caused oxidative damages in both cultivars. The intensity of H_2_O_2_, and consequently MDA, increased with the intensity of stress. Møller et al. [57] suggest that the higher MDA content caused more serious oxidative damage. This is evident in our study where commercial cultivar “Golden Delicious Reinders” possessed higher increased rate in the contents of MDA and H_2_O_2_ and earlier significant changes of H_2_O_2_ compared to the traditional cultivar “Dugara”, suggesting that “Dugara” possessed better drought tolerance. Previous studies also showed an increased level of MDA and H_2_O_2_ in olive and poplar plants under drought stress [58,59]. Content of MDA under drought stress in traditional cultivar “Crvenka” remained stable, while the content of H_2_O_2_ decreased at 12 day of drought. The finding of lower content of H_2_O_2_ under drought stress was in accordance with previous report of Umar and Shaheed-Siddiqui [60]. They presumed that the lower H_2_O_2_ production in drought stress might be due to the activation of antioxidant enzyme activities, particularly CAT, which detoxify H_2_O_2_ and reduces H_2_O_2_ accumulation.

Water content in leaves (WC) is an indicator of water status in plants used for drought tolerance estimation [61]. In response to drought stress conditions after (12 DAS), significant drops in water content (WC) were observed only in the leaves of the “Golden Delicious Reinders”, cultivar with the lowest photosynthetic efficiency. Traditional cultivars had better photosynthetic efficiency and unchanged water content. A study by Tounekti et al. [62] also showed that drought tolerant coffee cultivar had higher water content in the leaves with higher photosynthetic efficiency. All of these indicators support the data obtained in photochemical parameters, indicating better drought tolerance of traditional cultivars.

Proline is an important organic solute that is accumulated and increased in plants under drought conditions [63], acting as an ROS scavenging molecule [64]. Much previous research reported that drought tolerant plants had higher accumulation of proline under drought stress conditions [65,66]. In contrast to these findings, our results showed that proline measurements are not a reliable screening method for drought tolerance of investigated apple cultivars. The proline content was not higher in the drought tolerant cultivar “Crvenka” compared to drought sensitive “Golden Delicious Reinders”. Decreases in proline content in “Crvenka” indicate on activation some other defence mechanism against drought. Similar results were observed in the research of Rampino et al. [67] where drought tolerant wheat plants had higher relative water content (RWC) and reduced accumulation of proline. Plant phenolic compounds are secondary metabolites that act as antioxidants; they have been describe as indicators of abiotic stress tolerance in plants [68,69]. Hura et al. [70] found that triticale plants with better photosynthetic activity had higher levels of phenolic compounds. This is not consistent with our study where the amount of total phenolic compounds accumulated in the leaves of studied cultivars was very variable and the highest phenolic content was observed in the commercial cultivar “Golden Delicious Reinders”. These results suggest that phenols do not play a significant role in the defensive reactions of investigated apple cultivars. Similarly, Puente-Garza et al. [71] noticed that total phenolic content is not related to antioxidant activity in Agave plants. Although we hypothesized that higher concentrations of proline and secondary metabolites (phenols) will improve osmotic adjustment and tolerance to drought stress, this hypothesis is not supported, implying that in this study probably antioxidant enzyme systems were provoked as a defensive mechanism.

Our study concludes that specific differences in physiological and biochemical responses to drought stress among cultivars existed. Among the investigated cultivars, the more drought tolerant characteristic was observed in the traditional cultivar “Crvenka”. Higher photosynthetic efficiency, higher content of chlorophyll and greater membrane stability indicate that traditional cultivar “Crvenka” has the highest drought tolerance, while in contrast, the commercial cultivar “Golden Delicious Reinders” was the most sensitive in the investigated condition. Good quality traits of “Crvenka” [72] and good drought tolerance make this cultivar a promising candidate for cultivation under drought conditions in this area. These cultivars can be recommended for the revitalization of the production assortment in the study area and contribute to fruit growing development. The preservation of traditional cultivars is very important in order to preserve the genetic material for breeding; thus, the results from this study may provide valuable information for the future breeding programmes.

## 4. Materials and Methods

### 4.1. Apple Cultivars, Growth Conditions and Experimental Setup

The experiment was conducted in the greenhouse of Agricultural Institute Osijek, Croatia, in the summer (July 2019). Three apple (*Malus domestica* Borkh) cultivars were used in the experiment (“Golden Delicious Reinders”, “Crvenka” and “Dugara”). “Golden Delicious Reinders” is a widespread and very common commercial cultivar grown in intensive apple orchard systems. “Crvenka” and “Dugara” are traditional cultivars, originating from continental Croatia, grown mostly in small orchards. In the Balkan region, there are also many different names amongst those apple cultivars with the same genetic origin. One-year-old apple trees, grafted into rootstock M9, were grown in 25 L pots (one plant per pot) filled with soil (65% white peat, 35% black peat, 150 L clay/m^3^, 1500 g nitrogen-phosphorous-potassium fertilizer/m^3^). All the potted plants were regularly irrigated for 3 months under greenhouse conditions before drought stress was imposed. In this experiment drought stress was imposed by withdrawing irrigation. Five plants of each cultivar were under water-deficit (non-irrigated plants) and used as drought treatment, while five plants were optimally irrigated every day (2 L of water per day) and used as a control. The greenhouse’s mean day/night temperatures were 28 °C/20 °C at 7 DAS (day after stress) and 25 °C/15 °C at 12 DAS (day after stress), mean relative air humidity was 60/80% and 70/80%. The irradiance in the greenhouse was natural.

### 4.2. Measurement of Chlorophyll a Fluorescence

The measurements of chlorophyll *a* fluorescence were determined on leaves after 7 and 12 days of drought treatment. The measurements were performed on five plants per treatment for each cultivar on three fully developed leaves. The same leaf samples were collected, frozen in liquid nitrogen and stored at −80 °C for further analysis. Measurements were conducted on a sunny day, in the morning (8 h to 9:30 h) before the midday depression of photosynthesis. Chlorophyll *a* fluorescence was measured by a Handy-PEA fluorimeter (Plant Efficiency Analyser, Hansatech Instruments Ltd., Great Britain), which measures changes in chlorophyll *a* fluorescence during 10 μs to 1 s. Before measurement, all of the leaves were dark adapted using special leaf clips. After a dark-adapted period (30 min), leaves were exposed to a saturating red light pulses (wavelength in peak at 650 nm, 3.200 μmol (photon) m^–2^ s^–1^) which generate a fast fluorescence rise from the initial fluorescence intensity (F_0_) to a maximal intensity (F_m_). Fluorescence intensity was measured at 50 µs, when all PS II RCs are open (the O step) (F_0_); 100 µs; 300 µs; 2 ms (F_J_) (the J step); 30 ms (F_I_) (the I step) and 1 s maximal intensity when all PSII RCs are closed (F_m_ = P step). Recorded fluorescence values were used to calculate various parameters of the JIP test. The JIP test and analysis explore changes in photosystem II (PSII) photochemical performance, and it has been widely used as a measure of plants sensitivity to different stresses. It provides information about absorbed light energy as well as information on photosynthetic apparatus structure and function [73]. The parameters used for quantifying PSII behavior are the absorbed energy flux (ABS/RC), trapped energy flux (TR_0_/RC), electron transport flux (ET_0_/RC), dissipated energy flux (DI_0_/RC), the efficiency with which an electron can move from the reduced intersystem electron acceptors to the PS I end electron acceptors (RE_0_/ET_0_), performance index (PI_ABS_), performance index for energy conservation from exciton to the reduction of PSI end acceptors (PI_total_) and maximum quantum yield of primary photochemistry (TR_0_/ABS; F_v_/F_m_). Calculations are shown in Table 1.

Chlorophyll fluorescence transient data were demonstrated as OJIP curves and plotted on a logarithmic scale from 50 µs to 1 s, where O, J, I, P steps were marked. The OJIP transients were double normalized between O and P steps: W_OP_ = (F_t_ − F_0_)/(F_P_ − F_0_). For further analysis of drought-induced changes in the OJIP curve and to evaluate differences between control plants and drought-stressed plants of each cultivar, the differential curves were presented separately. For visualization of L-band, fluorescence data were normalized between O (50 μs) and K (300 μs) steps, as V_OK_ = (F_t_ − F_0_)/(F_K_ − F_0_) and plotted as difference kinetics ΔV_OK_ = V_OK(drought)_ − V_OK (control)_. For visualization of the K-band, fluorescence data were normalized between O and J (2 ms) steps as W_OJ_ = (F_t_ − F_0_)/(F_J_ − F_0_) and plotted as difference kinetics ΔW_OJ_ = V_OJ (drought)_ − V_OJ (control)_ [40,73].

### 4.3. Analysis of Total Chlorophyll Content and Carotenoids

After chlorophyll *a* measurements, a bulk sample of the same fifteen leaves per cultivar was used for the analyses of total chlorophyll content (Chl *a + b*) and carotenoids (Car). The leaves were homogenized into a fine powder using liquid nitrogen with the addition of magnesium hydroxide carbonate. Photosynthetic pigments in the samples of leaves were extracted and re-extracted seven times with absolute acetone until it was completely uncolored to obtain maximum pigment utilization in five repetitions. Absorbance of the solution was measured by spectrophotometer (Specord 200, Analytik, Jena, Germany) at 470, 647 and 663 nm. The concentrations of chlorophyll *a* (Chl *a*), chlorophyll *b* (Chl *b*) and carotenoids (Car) were calculated according to the equations of Lichtenthaler [74] and expressed as milligram per gram of dry weight (mg/g DW).

### 4.4. Extraction and Determination of Lipid Peroxidation and Hydrogen Peroxide

Extraction was performed by 1 mL of 0.1% (*w/v*) trichloroacetic acid (TCA) per 0.20 g of tissue powder. After extraction in the ice bath, homogenates were centrifuged for 15 min at 12,000× *g* at 4 °C and supernatants were used for determination of lipid peroxidation and hydrogen peroxide.

Lipid peroxidation was measured as content of malondialdehyde (MDA) in the leaves by the method of Verma and Dubey [75]. A total of 0.5 mL of the supernatant was mixed with 1 mL of 0.5% thiobarbituric acid (TBA) in 20% trichloroacetic acid (TCA), and thereafter it was heated for 30 min at 95 °C in a heating block and then cooled on ice. After centrifugation for 15 min at 14,000× *g* at 4 °C, the absorbance of the supernatant was read at 532 and 600 nm. The content of MDA was estimated by using the extinction coefficient of 155 mM^−1^ cm^−1^, and concentration was expressed as nanomole per gram of fresh weight (nmol/g FW).

Hydrogen peroxide (H_2_O_2_) concentration was quantified according to Velikova et al. [76]. After the addition of 0.5 mL of supernatant into 10 mM potassium phosphate buffer (pH 7.0) (0.5 mL) and 1 M potassium iodide (1 mL), reaction mixture was stored in darkness for 20 min. Absorbance of the reaction mixture was read at 390 nm, and H_2_O_2_ content was determined using a calibration curve obtained with different concentrations of H_2_O_2_ and expressed as micromoles per gram of fresh weight (µmol/g FW).

### 4.5. Extraction and Determination of Total Phenolic and Proline Content

Extraction was performed by 80% ethanol per 0.10 g of tissue powder and kept overnight at 4 °C. After centrifugation at 14,000× *g* at 4 °C for 10 min, supernatants were used for determination of total phenolic and proline content.

Proline content was analysed by the procedure of Woodrow et al. [77]. Aliquots of 50 μL, as well as proline standards (in range of 0.2 to 1 mM) in 4:1 ethanol: water (*v/v*), were dispensed into reaction tubes. In each tube, we added 100 μL of a reaction mixture prepared with ninhydrin 1% (*v/v*) in acetic acid 60% (*v/v*) and ethanol 20% (*v/v*). The tube content was mixed and heated in a heating block at 95 °C for 20 min. After cooling at room temperature, tubes were centrifuged at 500× *g* for 1 min, and then 100 μL of the mixtures were transferred to polypropylene microplate. The absorbance at 520 nm was measured by an Epoch microplate spectrophotometer (Bio-Tek, Germany). Proline content was calculated according to obtained standard curve and expressed as micromoles per gram of fresh weight (μmol/g FW).

Total phenolic content was determined by modified Folin–Ciocalteu method [78]. A total of 20 µL of supernatant was mixed with 1.58 mL of dH_2_O and 0.1 mL of Folin–Ciocalteu reagent (1:1; *v/v* diluted with water). After 5 min, 0.3 mL of sodium carbonate solution (20%; *v/v* diluted with water) was added. Homogenized reaction mixture was placed for 30 min in a dark place at room temperature. Absorbance of the solution was measured by spectrophotometer (Specord 200, Analytik, Jena) at 765 nm. The content of total phenolic was expressed as milligrams of gallic acid equivalents (GAE) per g based on a gallic acid calibration curve.

### 4.6. Determination of Water Content

After measuring chlorophyll fluorescence, a fraction of the leaves were sampled from the apple cultivars grown under either control or drought conditions and weighed to obtain the leaf fresh weight (FW). Thereafter, the weighed fraction of leaves was dried in the oven at 75 °C and weighed again to determine dry weight (DW). The water content in the leaves was calculated based on the equation [79]:

WC (%) = (FW − DW)/FW × 100

### 4.7. Statistical Analysis

Analysis of variance was carried out to determine differences among the treatments and cultivars on the 7th and 12th day of drought treatment. Mean comparisons were performed using the least significant difference (LSD) test at a 0.05 level of probability. The results are presented as means ± standard error of fifteen replicates for Chl *a* fluorescence parameters; five replicates for chlorophyll, carotenoid, MDA, H_2_O_2_, proline and phenols content and three replicates for leaf water content.

## Figures and Tables

**Table 1:** Parameters and formulae used in the analysis of the OJIP fluorescence transient.

TR_0_/ABS: Maximum quantum yield of PSII; TR_0_/ABS = [1 − (F_0_/F_m_)]
ABS/RC: Absorption per active RC; ABS/RC = M_0_ (1/V_J_) [1/(F_v_/F_m_)]
TR_0_/RC: Trapping per active RC; TR_0_/RC = M_0_ (1/V_J_)
ET_0_/RC: Electron transport per active RC; ET_0_/RC = M_0_ (1/V_J_) (1 − V_J_)
DI_0_/RC: Dissipation per active RC; DI_0_/RC = (ABS/RC) − (TR_0_/RC)
RE_0_/ET_0_: The efficiency with which an electron can move from the reduced intersystem electron acceptors to the PS I end electron acceptors; (1 − V_I_)/(1 − V_J_)
PI_ABS_: Performance index; PI = (RC/ABS) (TR_0_/DI_0_) [ET_0_/(TR_0_ − ET_0_)]
PI_total_: Performance index for energy conservation from exciton to the reduction of PSI end acceptors;
PI_total_ = PI_ABS_ × [(RE_0_/ET_0_)/(1 − RE_0_/ET_0_)]

All fluorescence parameters are in relative units. RC—reaction center.

## Data Availability

All data are contained within the article.
